# Comparison of Deep Learning Models for LAI Simulation and Interpretable Hydrothermal Coupling in the Loess Plateau

**DOI:** 10.3390/plants14152391

**Published:** 2025-08-02

**Authors:** Junpo Yu, Yajun Si, Wen Zhao, Zeyu Zhou, Jiming Jin, Wenjun Yan, Xiangyu Shao, Zhixiang Xu, Junwei Gan

**Affiliations:** 1 College of Resources and Environment, Yangtze University, Wuhan 430100, China; junpo_yu@163.com (J.Y.); zeyu_zhou@nwafu.edu.cn (Z.Z.); shaoxiangyu200107@163.com (X.S.); 19871829286@163.com (Z.X.); 18279571608@163.com (J.G.); 2 College of Water Resources and Architectural Engineering, Northwest A&F University, Yangling 712100, China; yajunsi@nwafu.edu.cn; 3 Lanzhou Institute of Arid Meteorology, China Meteorological Administration/Key Laboratory of Arid Climate Change and Reducing Disaster of Gansu Province/Key Open Laboratory of Arid Climate Change and Reducing Disaster, China Meteorological Administration, Lanzhou 730020, China; gstszhwe@163.com (W.Z.); dhywenjun@126.com (W.Y.)

**Keywords:** LAI, deep learning, water–heat coupling, vegetation dynamics simulation, Loess Plateau

## Abstract

As the world’s largest loess deposit region, the Loess Plateau’s vegetation dynamics are crucial for its regional water–heat balance and ecosystem functioning. Leaf Area Index (LAI) serves as a key indicator bridging canopy architecture and plant physiological activities. Existing studies have made significant advancements in simulating LAI, yet accurate LAI simulation remains challenging. To address this challenge and gain deeper insights into the environmental controls of LAI, this study aims to accurately simulate LAI in the Loess Plateau using deep learning models and to elucidate the spatiotemporal influence of soil moisture and temperature on LAI dynamics. For this purpose, we used three deep learning models, namely Artificial Neural Network (ANN), Long Short-Term Memory (LSTM), and Interpretable Multivariable (IMV)-LSTM, to simulate LAI in the Loess Plateau, only using soil moisture and temperature as inputs. Results indicated that our approach outperformed traditional models and effectively captured LAI variations across different vegetation types. The attention analysis revealed that soil moisture mainly influenced LAI in the arid northwest and temperature was the predominant effect in the humid southeast. Seasonally, soil moisture was crucial in spring and summer, notably in grasslands and croplands, whereas temperature dominated in autumn and winter. Notably, forests had the longest temperature-sensitive periods. As LAI increased, soil moisture became more influential, and at peak LAI, both factors exerted varying controls on different vegetation types. These findings demonstrated the strength of deep learning for simulating vegetation–climate interactions and provided insights into hydrothermal regulation mechanisms in semiarid regions.

## 1. Introduction

Vegetation serves as a critical biophysical regulator of land–atmosphere interactions, mediating fundamental processes including surface energy partitioning, hydrological cycling, and carbon sequestration [1,2]. As a key biophysical parameter of vegetation, Leaf Area Index (LAI) quantifies vegetation structure and its functional capacity. It directly influences canopy-level photosynthetic rates and stomatal regulation, playing a primary role in shaping ecosystem–climate feedbacks [3]. In agricultural ecosystems, for example, changes in LAI could be linked to crop yields and irrigation water use efficiency [4]. Despite its ecological significance, large-scale LAI modeling continues to face substantial challenges.

Current land surface models encounter two key limitations in LAI simulation. First, static parameterization schemes, such as fixed lookup tables, cannot capture dynamic, climate-driven vegetation responses [5,6]. Second, although dynamic vegetation modules incorporate biophysical processes such as photosynthesis and respiration, they often produce notable biases due to the complexity of land surface interactions [7,8]. To address these limitations, conventional efforts to improve LAI simulation have mainly relied on two methods, namely data assimilation and dynamic vegetation modules. Data assimilation integrates satellite-based LAI observations, such as Moderate Resolution Imaging Spectroradiometer (MODIS), into model outputs, which helps to reduce short-term biases [9]. However, this strategy depends heavily on historical data and fails to overcome the fundamental limitations of dynamic vegetation modeling [10]. In contrast, dynamic vegetation modules strive to improve simulation accuracy by introducing additional parameters, yet they often require input data that are overly complex [11]. This issue is particularly evident in crop modules, which must explicitly account for agricultural management practices [12]. Together, these challenges create significant obstacles for coupling dynamic vegetation growth models with ecosystems [13].

In recent years, deep learning methods have shown strong performance in meteorological and hydrological studies. For example, large-scale deep learning models have outperformed traditional numerical models in short-term weather forecasting [14,15] and have also been used to analyze the impacts of global warming on hydrological variability [16]. In the context of LAI modeling, various deep learning techniques have been explored, such as using Long Short-Term Memory (LSTM) networks and attention mechanisms to capture temporal dynamics [17,18,19], and integrating deep learning with remote sensing-based crop models to improve monitoring of crop growth [20]. These advances indicate that deep learning can address some disadvantages of empirical approaches in traditional numerical models, thus supporting more detailed simulations of vegetation and agricultural systems. However, a critical challenge remains that the opaque nature of deep learning models limits the interpretation of how hydrometeorological factors affect vegetation dynamics.

Against this backdrop, this study chose two key variables influencing vegetation growth, e.g., soil moisture and temperature. Soil moisture directly determines water availability, which can affect phenological dynamics and vegetation development [21,22,23]. Temperature influences vegetation growth by controlling the onset and duration of the growing season and restricting some key physiological processes [24]. This study had two primary objectives. First, we aimed to evaluate and compare the performance of three deep learning models, namely Artificial Neural Network (ANN), Long Short-Term Memory (LSTM), and Interpretable Multivariable (IMV)-LSTM, in simulating dynamic Leaf Area Index (LAI) for various vegetation types across the Loess Plateau, north–central China. These models utilized soil moisture and temperature as key input variables. Second, leveraging the feature weight matrices from the IMV-LSTM, we sought to quantify and interpret the changing relative importance of soil moisture and temperature in driving LAI dynamics for different vegetation types, providing insights into their impacts under varying conditions.

## 2. Materials and Methods

### 2.1. Study Area

**Figure 1:**
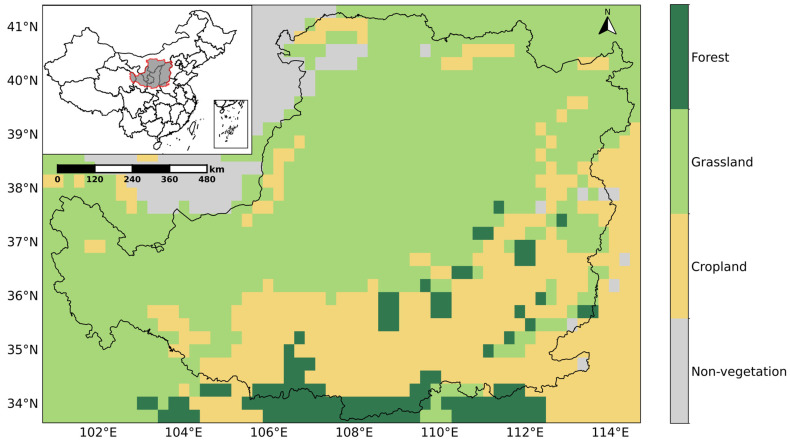
Location and land cover types in the Loess Plateau. The land cover types are derived from the International Geosphere-Biosphere Programme classification data in the MCD12Q1 product [34].

This study focused on the Loess Plateau in China (33°43′ N–41°16′ N, 100°54′ E–114°33′ E), a region characterized by an average elevation of between 800 and 1500 m above sea level, high gully density, high erodibility of the loess, low vegetation cover, and historical overexploitation [25], which covers an area of approximately 640,000 km^2^ [26] (Figure 1). This region experiences a temperate continental climate, with annual precipitation (average temperature) ranges from 300 mm (4.3 °C) in northwest to 700 mm (14.3 °C) in southwest [27,28]. Most of the area is classified as semi-arid due to these climatic conditions. During the past decades, this region experienced severe soil erosion, rendering its ecological environment fragile and highly sensitive to climate change and human activities [29,30]. Limited precipitation and relatively large fluctuations in temperature, together with agricultural activities, result in sparse vegetation in the Loess Plateau. Consequently, grasslands are the dominant vegetation type in this region, followed by farmlands and forests, while human settlements and desert areas constitute the primary non-vegetated zones. In addition, the vertical joint system formed by the Cenozoic tectonic movements results in a groundwater recharge rate of less than 5% of annual precipitation, making soil moisture a key controlling factor in the soil–plant-atmosphere continuum in the Loess Plateau [31,32,33]. Therefore, these characteristics of the Loess Plateau provide favorable conditions to investigate the controlling effects of soil moisture and temperature on vegetation.

### 2.2. Datasets and Preprocessing

#### 2.2.1. Meteorological Data

The Global Land Data Assimilation System (GLDAS) provides global land surface state variable data at a 3 h temporal resolution (from 2000 to present) and a 0.25° spatial resolution [6]. In this study, we focused on the two most important climatic factors influencing vegetation productivity, namely, soil moisture and temperature [35,36]. The soil moisture data from GLDAS are available for four distinct layers (0–10 cm, 10–40 cm, 40–100 cm, and 100–200 cm). Here, we used the cumulative soil moisture content from the top three layers (total depth of 100 cm) as our input variable, which encompasses the main rooting depth of most vegetation while avoiding excessive depth affecting the simulation of shallow-rooted vegetation [37].

#### 2.2.2. LAI Data

In this study, we used the reprocessed MODIS LAI C6.1 dataset, covering the period from 2000 to 2021 [38]. The original MODIS LAI values are produced using two algorithms. The primary algorithm is based on three-dimensional radiative transfer theory. The backup algorithm uses the relationship between the Normalized Difference Vegetation Index (NDVI) and LAI [39]. However, such methods can introduce uncertainties due to atmospheric interference, sensor limitations, and retrieval algorithm errors, often leading to data inconsistencies and noise. The reprocessed dataset applies the modified temporal spatial filter method and Savitzky–Golay filtering [36,40], improving the temporal continuity and spatial consistency of the data. We used the 8-day temporal resolution and 0.25° spatial resolution to match the meteorological data.

#### 2.2.3. Land Cover Type Data

The land cover data were derived from the MODIS Landcover MCD12Q1 dataset, including landcover information under multiple classification schemes [34]. This dataset provides annual land use types from 2001 to 2020. To provide a consistent background for our study period (2000–2021) and to minimize the impact of inter-annual land cover variability on our primary analysis of LAI dynamics, we exclusively used the 2010 land cover data, which is considered representative of the overall study period. Additionally, we used the International Geosphere-Biosphere Programme classification scheme, which involves a total of 17 land cover classes. Among these classes, nine land cover types are present in the Loess Plateau. To simplify analysis and reduce complexity, these nine classes were grouped into four aggregated land cover types, which are forests, grasslands, farmlands, and non-vegetation areas (Figure 1). Specifically, deciduous broad-leaved forests and mixed forests were combined as forests; savannas, grasslands, and meadows were aggregated into grasslands; farmlands and farmland–natural vegetation mosaics were merged as farmlands; urban and built-up areas together with deserts were classified as non-study areas.

#### 2.2.4. Data Preprocessing

To ensure spatiotemporal consistency across multi-source datasets, we implemented systematic preprocessing procedures. Temporally, the original three-hourly soil moisture and temperature measurements were first averaged to daily values, and these daily values were then aligned with LAI data by date and subsequently resampled to match the 8-day resolution of the LAI data for temporal synchronization. Spatially, soil moisture, temperature, and LAI were preprocessed to avoid inconsistency such as possible misalignment at land–sea boundaries. Specifically, all datasets were regridded to a common grid, and only those grids with valid values in each dataset were retained. Furthermore, grids with an average LAI below 0.1 throughout the entire time series were considered invalid and excluded from analysis. This threshold was chosen because our analysis showed that LAI values below 0.1 exhibited clear anomalies and such grids constituted just 0.04% of all valid grids.

To address differences in variable scales, all variables were normalized using the min–max method. The minimum and maximum ranges for each variable were defined below. Soil moisture values were constrained between 0 and 1000 kg/m^2^, temperature spanned from −50 to 50 °C, and LAI was bounded between 0 and 10 m^2^/m^2^.

Considering the temporal coverage of these three datasets, the study period was determined for the period of 2000 to 2021. For each grid, soil moisture and temperature were temporally aligned and concatenated, and a moving window of seven consecutive time steps was extracted as input sequence. The temporal resolution of each time step matches that of the LAI data (8 days), and thus the moving window covers a total period of 56 days. This window duration was chosen based on sensitivity tests that optimized model performance while considering computational efficiency, and is consistent with findings in the literature indicating that the lagged effects of meteorological variables on vegetation growth typically extend for approximately two months [41,42]. The LAI value corresponding to the last time step in each window served as the prediction target. This means that each sample contains soil moisture and temperature from seven time steps as input, with one LAI value at the final time step as the target. This window was moved forward by one time step along the time series (2000–2021), repeating the process until the entire time series was covered. The final dataset used for training encompasses 1870 grid cells within the Loess Plateau region. For each grid cell, a total of 1012 samples were generated. Each sample comprises two input variables (soil moisture and air temperature) and one target variable (LAI). Each input variable consists of data from seven time steps, with an 8-day resolution per time step, resulting in a 56-day temporal span for each input variable. The target variable, LAI, is represented by a single value corresponding to the last day of the input sequence.

### 2.3. Model Architecture

In this study, three models were used for LAI simulations, namely ANN, LSTM, and IMV-LSTM. The ANN model is a fully connected neural network with a multilayer perceptron (MLP) architecture, which can approximate any function given a sufficiently large number of parameters according to the universal approximation theorem [43]. The LSTM model, as a type of gated recurrent neural network, introduces temporal dependencies via its recurrent structure, thereby enabling effective modeling of time series data [44]. However, both ANN and LSTM are considered black box models, which makes it challenging to interpret the influence of input features on the modeling process. To address this limitation, we introduced the IMV-LSTM model, which incorporates an attention mechanism to weight the LSTM output features [45]. This approach enables dynamic assessment of the varying importance of input features throughout the simulation process. The fundamental principles of each model used in this study are outlined below.

#### 2.3.1. Artificial Neural Network·

The computation in each layer of ANN is expressed as follows:

(1) $$h^{(l)\;}=\;\tan h\left(W^{(l)T}h^{\left(l-1\right)}+b^{\left(l\right)}\right)\;\;\;\;(l\;=\;1,\ldots ,L)$$

where $h^{\left(l\right)}\in R^{n_{l}}$ is the output vector of the l-th hidden layer; $n_{l}$ is the number of neurons in this layer; $W^{\left(l\right)T}\in R^{n_{l}\times n_{l-1}}$ denotes the weight matrix connecting the $\left(l-1\right)$-th and l-th layers; $b^{\left(l\right)}\in R^{n_{l}}$ is the bias vector for the l-th layer. The network consists of four hidden layers and one output layer. The tanh function is used as the activation function in the hidden layers. The input data were first flattened into one-dimensional arrays and fed into the model all at once, resulting in a single LAI value as output.

#### 2.3.2. Long Short-Term Memory

The main components and operations of LSTM are summarized below:

(2) $$i_{t}=\;\sigma \left(W_{i}\cdot \left[h_{t-1},x_{t}\right]+b_{i}\right)$$

(3) $$f_{t}=\sigma \left(W_{f}\cdot \left[h_{t-1},x_{t}\right]+b_{f}\right)$$

(4) $$o_{t}=\sigma \left(W_{o}\cdot \left[h_{t-1},x_{t}\right]+b_{o}\right)$$

where $i_{t},f_{t},o_{t}$ are the update gate, forget gate, and output gate at time step t, respectively. The vector $x_{t}\in R^{d}$ is the input feature vector at time t, and $h_{t}\in R^{h}$ is the hidden state vector. Next, the candidate memory cell, the updated memory cell, and the updated hidden state are computed in the following:

(5) $${\tilde{C}}_{t}=\;tanh\left(W_{c}\cdot \left[h_{t-1},x_{t}\right]+b_{c}\right)$$

(6) $$C_{t}=f_{t}*C_{t-1}+i_{t}*{\tilde{C}}_{t}$$

(7) $$h_{t}=o_{t}*tanh\left(C_{t}\right)$$

where $C_{t}\in R^{h}$ is the memory cell vector at time t, and ${\tilde{C}}_{t}\in R^{h}$ is the candidate vector for the memory cell. This model received input data from only one time step at a time and processed the sequence step by step. The prediction was produced using the output corresponding to the final time step.

#### 2.3.3. Interpretable Multivariable Long Short-Term Memory

The IMV-LSTM is based on the IMV-Tensor architecture, which uses the tensor operations to maintain variable-level feature representations and to enhance interpretability with a dual-stage attention mechanism [45]. The key tensor computation is defined as follows:

(8) $${\tilde{j}}_{t}=\tan h({𝓦}_{j}⊛{\tilde{h}}_{t-1}+{𝓤}_{j}⊛x_{t}+b_{j})$$

where ${\tilde{h}}_{t}={\left[h_{t}^{1},\ldots ,h_{t}^{N}\right]}^{T}$ and ${\tilde{h}}_{t}\in R^{N\times d}$. N is the number of input variables, and $h_{t}^{n}\in R^{d}$ is the hidden state vector of the n-th input variable at time t. The matrix ${𝓤}_{j}={\left[U_{j}^{1},\ldots ,U_{j}^{N}\right]}^{T}$, where ${𝓤}_{j}\in R^{N\times d\times d_{0}}$ and $U_{j}^{n}\in R^{d\times d_{0}}$. $d_{0}$ is the dimension of each input variable at one time step. The transition matrix is ${𝓦}_{j}=\left[W_{j}^{1},\ldots ,W_{j}^{N}\right]$, where ${𝓦}_{j}\in R^{N\times d\times d_{0}}$ and ${𝓦}_{j}^{n}\in R^{d\times d_{0}}$. The output ${\tilde{j}}_{t}={\left[j_{t}^{1},\ldots ,j_{t}^{N}\right]}^{T}$ has the same shape, where $j_{t}^{n}\in R^{d}$ corresponds to the update of the hidden state for variable n.

The update equations for hidden state and cell state based on tensors are as follows:

(9) $$\left[\begin{array}{c} {\tilde{i}}_{t}\\ {\tilde{f}}_{t}\\ {\tilde{o}}_{t} \end{array}\right]=\;\sigma \left(𝓦⊛{\tilde{h}}_{t-1}+\;𝓤⊛x_{t}+\;b\right)$$

(10) $${\tilde{c}}_{t}={\;\tilde{f}}_{t}⊙{\tilde{c}}_{t-1}+{\tilde{i}}_{t}⊙{\tilde{j}}_{t}$$

(11) $${\tilde{h}}_{t}={\tilde{o}}_{t}⊙\tan h({\tilde{c}}_{t})$$

where ⊛ is defined as the product of two tensors along the N axis, and ⊙ denotes element-wise multiplication.

The hybrid attention layer integrates the IMV-Tensor output into two stages. First, the variable-specific hidden state sequences are passed to the temporal attention module, which captures the temporal information for each variable over the entire sequence. Next, the resulting hidden states, including all historical information, are sent to the variable attention module. This module combines information from all variables and generates a weight matrix. The elements in this matrix range from 0 to 1 and estimate the relative importance of each variable. The final output is a weighted result based on this matrix.

The hybrid attention mechanism is formulated as follows:

(12) $$p\left(y_{T+1}|X_{T}\right)=\sum_{n=1}^{N}p(y_{T+1}|z_{T+1}=n,h_{T}^{n}⊕g^{n})\cdot Pr(z_{T+1}=n|h_{T}^{1}⊕g^{1},\ldots ,h_{T}^{N}⊕g^{N})$$

(13) $$g^{n}=\sum_{t=1}^{T}\alpha_{t}^{n}$$

(14) $$\alpha_{t}^{n}=\frac{\exp\left(f_{n}\left(h_{t}^{n}\right)\right)}{\sum_{k=1}^{T}\exp\left(f_{n}\left(h_{k}^{n}\right)\right)}$$

where $z_{T+1}$ is a discrete variable that takes values from 1 to N and controls the density function of $y_{T+1}$; N is the number of input variables; $p\left(y_{T+1}|z_{T+1}=n,h_{T}^{n}⊕g^{n}\right)$ is the conditional density of $y_{T+1}$ when variable n plays the leading role; ⊕ denotes the concatenation; $Pr\left(z_{T+1}=n|h_{T}^{1}⊕g^{1},\ldots ,h_{T}^{N}⊕g^{N}\right)$ gives the weight for variable n. This weight reflects the contribution of variable n to the prediction. $g^{n}$ is the attention weighted sum of the hidden states for variable n over all time steps. The attention weight is calculated as $\alpha_{t}^{n}$, where $f_{n}$ is a variable-specific scoring function that is learned during training. This model processes inputs in the same way as an LSTM. However, rather than relying solely on the output at the last time step, it leveraged the outputs from all time steps of the LSTM layer. These were subsequently processed by a hybrid attention mechanism to generate the final prediction.

### 2.4. Experimental Setup

A dataset covering 22 years (2000–2021) was used in our experiments. The dataset was partitioned chronologically into training (70%, ~15 years), validation (10%, ~2 years), and testing subsets (20%, ~4 years). During training, the Adam optimizer [46] was used with a learning rate of 0.001. A batch size of 256 was employed, and each model was trained for 300 epochs. To select the most robust model and mitigate overfitting, only the model weights corresponding to the minimum validation loss over 300 epochs were retained, without employing any additional regularization methods. This approach effectively served as an early stopping strategy for model selection. The ANN model included four hidden layers (32, 256, 256, and 32 neurons, respectively) and a single-neuron layer for output. Both the LSTM and IMV-LSTM models used a single LSTM layer with 256 hidden units and the number of neurons in attention layer was determined by the LSTM layer. Training data from all valid global grids were merged to form a global pre-training dataset. The model was pre-trained on this dataset to learn general relationships across different climatic and vegetative settings, and then it was fine-tuned on each grid to capture local surface heterogeneity. These processes thus produced a unique set of model parameters for each grid.

### 2.5. Statistical Analysis

In this study, we used the coefficient of determination (R^2^), root mean squared error (RMSE), and mean absolute error (MAE) to evaluate the model performance. The definitions of these metrics are as follows:

(15) $$R^{2}=\;1-\frac{\sum_{i}{\left(y_{i}-{\hat{y}}_{i}\right)}^{2}}{\sum_{i}{\left(y_{i}-\bar{y}\right)}^{2}}$$

(16) $$RMSE=\sqrt{\frac{\sum_{i}{\left(y_{i}-{\hat{y}}_{i}\right)}^{2}}{K}}$$

(17) $$MAE=\frac{\sum_{i}\left|y_{i}-{\hat{y}}_{i}\right|}{K}$$

where $y_{i}$ is the i-th observed value, ${\hat{y}}_{i}$ is the i-th predicted value, ${\bar{y}}_{i}$ is the mean of the observed value, K is the number of samples, and i∈{1,2,…,K}. Higher R^2^ and lower RMSE and MAE indicate that the observed data agree well with the predicted data.

## 3. Results

### 3.1. Model Evaluation

**Figure 2:**
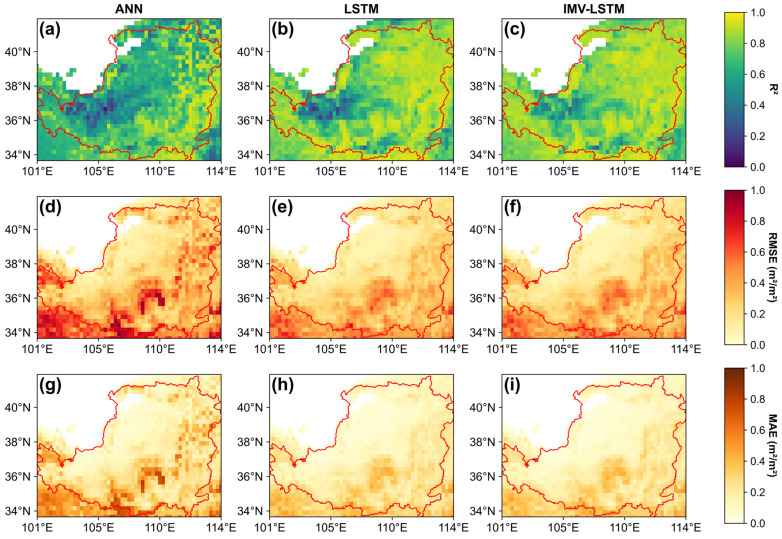
Spatial distributions of model evaluation metrics over the Loess Plateau. (**a**,**d**,**g**) represent ANN; (**b**,**e**,**h**) represent LSTM; (**c**,**f**,**i**) represent IMV-LSTM. (**a**–**c**) represent the coefficient of determination; (**d**–**f**) represent the root mean squared error; and (**g**–**i**) represent the mean absolute error.

Significant performance differences in LAI predictions were observed between the ANN and the time series models (LSTM and IMV-LSTM; Figure 2). The time series models consistently outperformed ANN, with IMV-LSTM achieving the highest overall accuracy. Specifically, the spatially averaged R^2^ of the whole Loess Plateau using IMV-LSTM was 0.817, surpassing ANN (0.682) and LSTM (0.794) by 19.8% and 2.9%, respectively. In terms of error metrics, IMV-LSTM also demonstrated superior performance, yielding the lowest RMSE (0.272 m^2^/m^2^), at 24.2% and 1.5% lower than ANN (0.359 m^2^/m^2^) and LSTM (0.276 m^2^/m^2^), respectively. Similarly, its MAE (0.189 m^2^/m^2^) was the smallest among these models, reflecting reductions of 29.5% and 1.6% compared to the ANN (0.268 m^2^/m^2^) and LSTM (0.192 m^2^/m^2^), respectively.

Further analysis revealed notable spatial differences in model performance across the region. Specifically, R^2^ decreased from northwest to southeast, and corresponding RMSE and MAE increased along the same gradient. Lower R^2^ was mostly observed in the arid northwest (annual precipitation < 400 mm) and some southern valleys, while higher RMSE and MAE appeared in central–southern and eastern forest regions with greater absolute LAI. Despite these spatial variations, the performance of IMV-LSTM remained robust where maximum RMSE did not exceed 0.8 m^2^/m^2^ and maximum MAE did not surpass 0.6 m^2^/m^2^ throughout the study area, confirming the model’s stability and adaptability under diverse environmental conditions. Collectively, IMV-LSTM demonstrated clear superiority over both LSTM and ANN in modeling LAI dynamics in the Loess Plateau.

### 3.2. LAI Preditions for Different Vegetations

**Figure 3:**
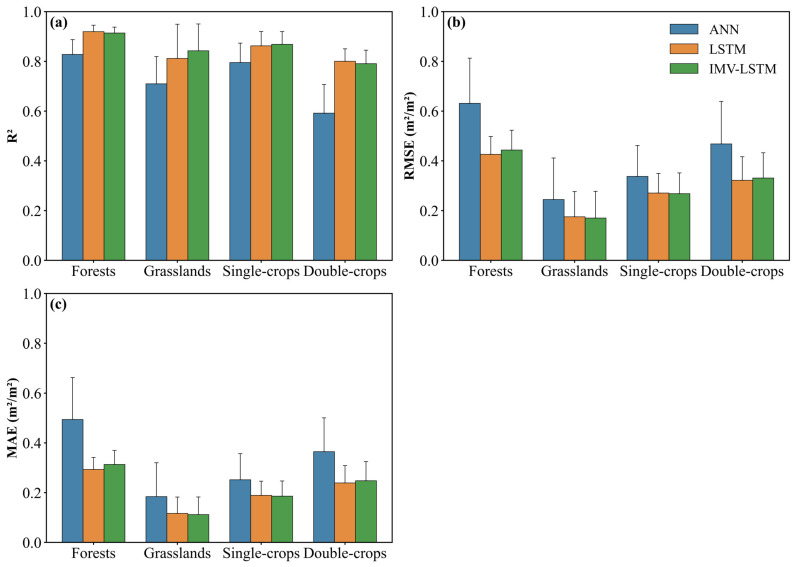
Model evaluation for distinct vegetation types with different models, and the error bars are standard deviation. (**a**) depicts the coefficient of determination, (**b**) displays the root mean squared error, and (**c**) presents the mean absolute error.

Time series models (IMV-LSTM and LSTM) also outperformed ANN in simulating LAI for diverse vegetation types (Figure 3). In this study, farmlands include two cropping systems, which are single-season crops and double-season rotation crops. Among all vegetation types, both the IMV-LSTM and LSTM models achieved the highest average R^2^ for forests (0.913 and 0.919, respectively), followed by single-season crops (0.868 and 0.862) and grasslands (0.842 and 0.812), and double-season rotation crops had the lowest R^2^ (0.791 and 0.800; Figure 3a). In comparison, the ANN model exhibited consistently lower R^2^ across all vegetation types. Its R^2^ scores were 0.828, 0.795, 0.709, and 0.592 for forests, single-season crops, grasslands, and double-season rotation crops. The greatest improvement was found in double-season crops, where accuracy increased by up to 35% compared to ANN, highlighting the benefit of time series structures in capturing complex seasonal dynamics. These results underscored the substantial advantage of LSTM-based time series approaches over the ANN model.

In addition to R^2^, error-based metrics were also used to assess the model performance (Figure 3b,c). All models attained the lowest RMSE and MAE for grasslands, followed by croplands, and forests yielded the highest bias. For example, RMSEs (MAEs) were 0.170 m^2^/m^2^ (0.112 m^2^/m^2^), 0.268 m^2^/m^2^ (0.186 m^2^/m^2^), 0.331 m^2^/m^2^ (0.248 m^2^/m^2^), and 0.444 m^2^/m^2^ (0.313 m^2^/m^2^) for grasslands, single-season crops, double-season crops, and forests, respectively, when using the IMV-LSTM model. However, ANN produced greater bias compared to those two time series models for all vegetable types. Specially, forests predictions using ANN showed substantially larger errors (RMSE = 0.631 m^2^/m^2^, MAE = 0.494 m^2^/m^2^) relative to time series modeling approaches.

**Figure 4:**
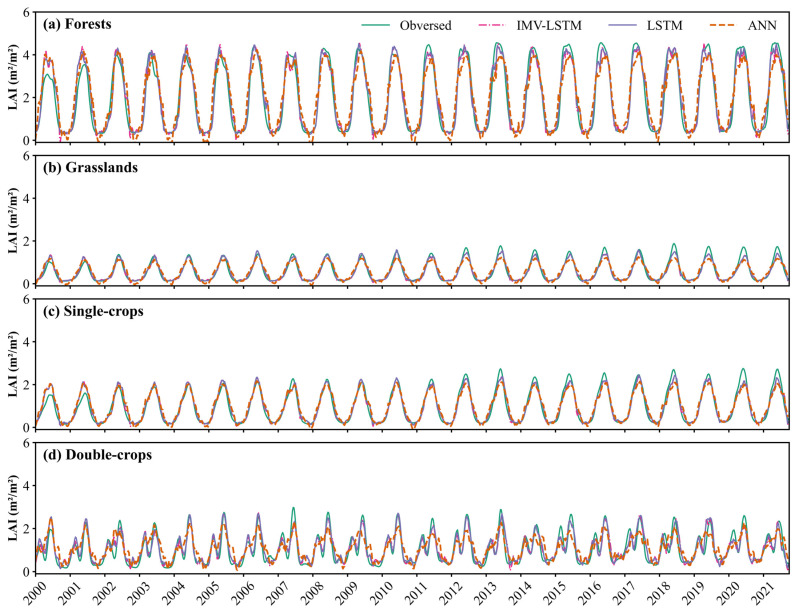
The average simulated LAI across different vegetation types in the temporal dimension with different models during 2000–2021. (**a**–**d**) correspond to forests, grasslands, single-season crops, and double-season rotation crops, respectively.

A comparative analysis of these models’ simulation performance was also conducted along the temporal dimension (Figure 4). For unimodal vegetation types such as forests, grasslands, and single-season crops (Figure 4a–c), all models produced relatively accurate simulations during both the increasing and declining phases of LAI, indicating their capacity to reflect seasonal dynamics. Nevertheless, systematic biases were still observed. During 2000–2001, all models consistently overestimated peak LAI values. From 2002 to 2010, time series models demonstrated accurate simulations of LAI peaks, while the ANN model systematically underestimated them. In contrast, during 2011–2021, all models exhibited a tendency to underestimate peak LAI. In addition, throughout the study period, the ANN model consistently underestimated minimum LAI, a pattern not observed with time series models. For bimodal croplands, represented by summer maize and winter wheat rotation, similar systematic errors as those observed for unimodal vegetation types were present (Figure 4d). The ANN model was unable to replicate the bimodal LAI characteristics of annual double-season rotation crops, instead often producing a unimodal pattern in the simulations. Even in years when partial bimodal features appeared, the transitions between the two peaks were unrealistic. In contrast, both LSTM and IMV-LSTM successfully reproduced the bimodal LAI structure, accurately capturing the abrupt decline and subsequent increase during the double-season growth cycle. These results highlighted the enhanced capability of time series models to represent the complex growth patterns in agricultural landscapes.

### 3.3. Variable Importance Assessment

**Figure 5:**
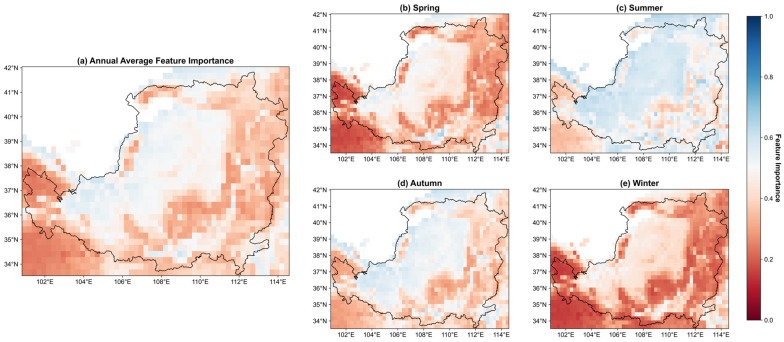
Spatial distribution of the relative importance of water and heat. (**a**) represents the average state of water–heat relative importance over all time, while (**b**–**e**) show their distribution patterns in the four seasons: spring, summer, autumn, and winter, respectively. Values greater than 0.5 indicate that soil moisture is more important than temperature (tending to blue), whereas values less than 0.5 signify temperature outweighs soil moisture in importance (tending to red).

The weight matrix produced by the hybrid attention layer of the IMV-LSTM model quantified how the relative importance of input variables changed dynamically during simulations, with values normalized to a range from 0 to 1. Because only soil moisture and temperature were included as input variables in this study, the analysis focused on the importance of soil moisture, with the importance of temperature calculated as one minus the importance of soil moisture. The importance of soil moisture greater than 0.5 indicated that soil moisture was dominant; otherwise, temperature exerted stronger influence. The results showed a clear geographical gradient in the regulation of vegetation growth by water and heat coupling across the Loess Plateau (Figure 5a). Soil moisture was the predominant driver in arid northwestern regions where annual precipitation was less than 400 mm. In contrast, temperature was the dominant factor in humid southeastern regions where annual precipitation exceeded 600 mm.

To further investigate the mechanisms underlying water and heat coupling in vegetation growth regulation in the Losses Plateau, we conducted analyses at the seasonal scales (Figure 5b–e). In spring (March to May), temperature-controlled areas covered 71% of this region (Figure 5b), but such areas narrowed to 28% in summer (June to August), while the remaining temperature-dominated zones correspond mainly to forests (Figure 5c). During autumn (September to November), temperature-controlled regions expanded as the influence of soil moisture declined in southeastern croplands and grasslands (Figure 5d). Forest ecosystems demonstrated a strong thermal regulation, with temperature control intensifying both in magnitude and spatial extent. In winter (December to February), nearly the entire Loess Plateau was controlled by temperature (Figure 5e), with the relative importance of temperature reaching a maximum value of 0.78 in forests, which was higher than in croplands (0.68) and grasslands (0.67).

**Figure 6:**
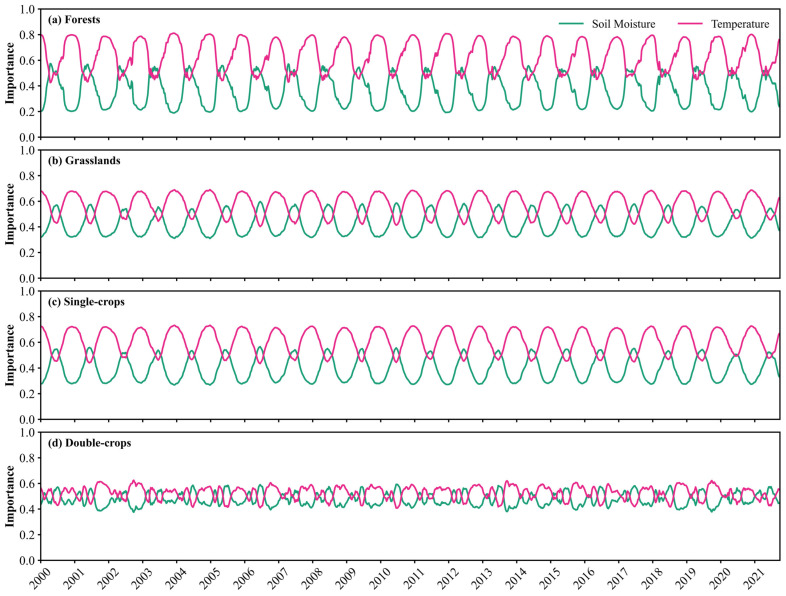
Temporal distribution of the relative importance of soil moisture and temperature across vegetation types. (**a**–**d**) correspond to forests, grasslands, single-season crops, and double-season rotation crops, respectively.

The relative importance of temperature versus soil moisture exhibited distinct temporal patterns across different vegetation types (Figure 6). Forest ecosystems exhibited the most pronounced variability, with temperature importance peaking at 0.80 while soil moisture importance reached a minimum of 0.20 (Figure 6a). Temperature remained the main controlling factor for most of the time, although soil moisture occasionally became more important for short periods. Grasslands and single-season crops displayed similar patterns (Figure 6b,c). In these ecosystems, temperature remained more important, but soil moisture continued to play a significant role for a longer duration compared to forests. Variability in grasslands and single-season crops was less pronounced, with temperature importance remaining below 0.7 and soil moisture above 0.3. However, unlike the consistent seasonal cycles observed in forests, grasslands, and single-season crops ecosystems, double-season crops exhibited irregular water–heat dynamics, characterized by multiple intra-annual peaks and significant inter-annual variability in temperature and moisture importance (Figure 6d).

**Figure 7:**
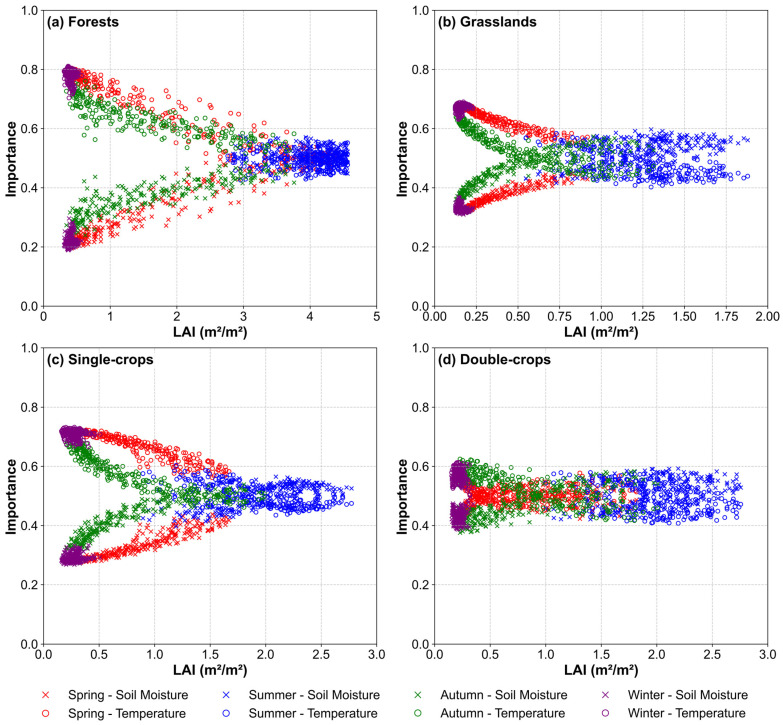
Relative importance of soil moisture and temperature as a function of LAI across vegetation types. (**a**–**d**) depict forests, grasslands, single-season crops, and double-season rotation crops, respectively. Spring, summer, autumn, and winter are denoted by red, blue, green, and purple, respectively. Soil moisture importance is indicated by cross markers, and temperature importance is shown by open circles.

Soil moisture and temperature are key drivers of vegetation growth, and their importance patterns shifted dynamically in relation to the changes in LAI (Figure 7). Notably, the importance of temperature exhibited a clockwise trend over time, whereas that of soil moisture showed a counterclockwise pattern. As LAI increased, the importance of temperature generally declined while that of soil moisture rose, reflecting vegetation’s increased water demand. However, the extent and pattern of this shift varied considerably among vegetation types. For forests, the importance of soil moisture and temperature converged nearly linearly throughout the growing season (Figure 7a). Both factors reached similar and balanced importance at the peak growth stage. For grasslands, the divergence between the importance of soil moisture and temperature emerged rapidly during early growth and remained relatively stable afterwards, with soil moisture clearly dominating at peak LAI and this gap being more pronounced compared to other vegetation types (Figure 7b). For a single-crop system, large fluctuations in the importance of each factor were evident during spring and autumn, whereas soil moisture became more important than temperature at the peak growth stage, with the gap being less pronounced than that in grasslands (Figure 7c). Unlike the previous three vegetation types, in the double-crop system, the difference between soil moisture and temperature importance remained small when the LAI was low, which was likely attributed to crop succession and agricultural interventions. Throughout the growing season, this difference stayed relatively minor and stable, and at peak growth, soil moisture was only slightly more important than temperature (Figure 7d).

**Figure 8:**
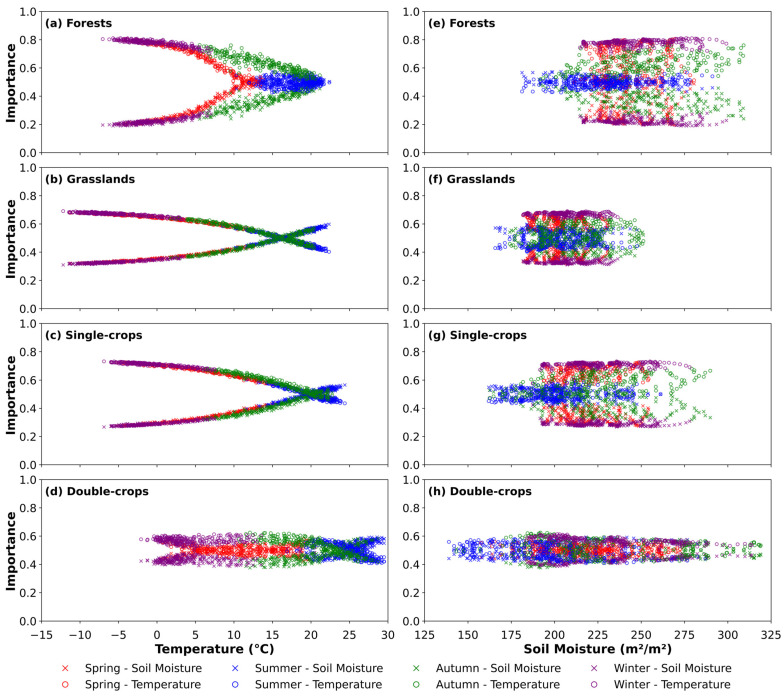
Seasonal variation in the importance of temperature and soil moisture across different vegetation types. (**a**–**d**) show the relationship between importance and temperature for forests, grasslands, single-season crops, and double-season crops, respectively. (**e**–**h**) show the relationship between predictor importance and soil moisture for the corresponding vegetation types. Colors and markers represent different seasons and variables, as indicated in the legend.

We further analyzed how the importance of temperature and soil moisture varied across different vegetation types (Figure 8). When temperature served as the independent variable, most vegetation types showed that the importance of temperature decreased as temperature increased, while the importance of soil moisture increased (Figure 8a–d). This contrasting trend appeared more strongly in forests, grasslands, and single-season crops. However, for double-season crops, the importance of soil moisture did not increase appreciably until temperature reached ~20 °C (Figure 8d). The temperature at which the importance of temperature and soil moisture became equivalent varied among vegetation types, occurring at ~16 °C in grasslands, ~20 °C in forests and single-season crops, and ~25 °C in double-season crops. When soil moisture acted as the independent variable, no clear pattern in variable importance emerged, unlike the results observed when temperature was used (Figure 8e–h).

## 4. Discussion

Time series models performed well for LAI simulations across different vegetation types in the Losses Plateau (Figure 2, Figure 3 and Figure 4), highlighting the significance of capturing temporal relationships in ecological modeling. Both LSTM and IMV-LSTM have higher R^2^ and lower RMSE and MAE values than ANN, which indicated that the time series models can better capture the lagged effects of climate on vegetation. In addition, we compared our results with previous studies that conducted LAI simulations based on the traditional physical models in the Losses Plateau [47,48]. Sun et al. used the Noah-MP model with seven meteorological variables to simulate LAI for the period of 2001 to 2018, producing a maximum R^2^ of 0.68 and an RMSE of 1.01 m^2^/m^2^ [47]. Similarly, the results in [48] indicated that all the R^2^ values for simulated LAI over the Loess Plateau using Noah, Community Land Model, and Simplified Simple Biosphere Model are below 0.5. Even after applying data assimilation techniques, the R^2^ only increases to around 0.7. Obviously, the time series models used in this study achieved the better performance, with the highest R^2^ and the lowest RMSE and MAE. Notably, LSTM substantially improved simulation performance for crops, especially for rotated crops, which have been considered challenging in an earlier study [17]. Furthermore, the interpretable architecture of IMV-LSTM enabled explicit quantification of soil moisture and temperature’s differential impacts on vegetation growth, providing mechanistic insights that facilitate further investigations.

The feature importance maps derived from the IMV-LSTM revealed clear spatiotemporal dynamics in how water and temperature regulate vegetation growth (Figure 5). Specifically, the dominant environmental factors varied markedly across regions. In the arid northwest, soil moisture emerged as the primary driver where annual precipitation is less than 400 mm. Conversely, in the humid southeast where annual precipitation exceeds 600 mm, temperature became the dominant factor influencing vegetation. This spatial variation can be attributed to the distinct limiting factors of different ecosystems. In water-limited regions, the nonlinear relationship between soil moisture and vegetation productivity has a direct impact on growth dynamics [49], whereas temperature is the key driver of phenological processes in heat-limited areas [50]. Interestingly, although temperature was the main control in the southeastern region, some areas with intensive cropland still showed soil moisture as the dominant factor, likely due to the high sensitivity of crop vegetation to water availability.

Seasonal variation further modulated these spatial patterns, as evidenced by the water and heat contribution maps (Figure 5b–e). In spring, temperature-controlled areas were lower than those in winter, indicating that rising temperatures reduced the limiting effect of heat (Figure 5b). During this period, soil moisture was more important in the arid northwest and southern valley croplands. In the northwest, grasslands with shallower root systems are more sensitive to fluctuations in temperature and soil moisture [51], and the increased water demand during winter wheat jointing, coupled with irrigation, accentuates the role of soil moisture in southern agricultural zones [52]. In summer, most areas were dominated by soil moisture (Figure 5c). However, a small portion of temperature dominated zones were mainly found in forests, which may be attributed to the fact that forests have deep root systems that allow them to access water from below the soil layer configured in this study [53,54]. In autumn, the distribution patterns were like those observed in spring, while the influence of soil moisture was more pronounced across the northwestern region (Figure 5d). In winter, temperature became the main factor controlling conditions in most areas (Figure 5e).

The relative importance of soil moisture and temperature as drivers of vegetation growth dynamically shifted with LAI changes (Figure 7). Seasonally, temperature mainly restricted vegetation growth during winter and the early spring (low LAI) for all vegetation types, but its constraint decreased as temperatures rose. The alleviation of thermal constraint promoted vegetation growth and increased water consumption, consequently elevating the relative importance of soil moisture availability [55]. In summer, soil moisture usually became the primary constraint for grasslands and crops, while both factors remained comparably important in forests. From autumn to winter, as temperatures fell, temperature importance rose again, plant physiological activities weakened, and LAI dropped, reducing the influence of soil moisture in all vegetation types [56]. These patterns underscored the distinct regulatory mechanisms by which temperature and soil moisture dynamically control LAI variations in the Loess Plateau.

Importance patterns also differed among vegetation types (Figure 7). Forest ecosystems exhibited significantly stronger temperature control over LAI dynamics compared to other vegetation types (Figure 7a). This predominance of thermal regulation likely stems from their deep root systems that mitigate water stress [53,54], thereby elevating temperature as the primary determinant. Grasslands exhibited stronger dependence on soil moisture availability compared to thermal regulation, particularly when temperature effects on vegetation growth became less pronounced (Figure 7b). Single-season crops behaved like grasslands but had weaker soil moisture signals (Figure 7c). This difference can be ascribed to the fact that native grasslands, evolved under moisture-limited conditions, maintain tight coupling with soil water availability, whereas agricultural systems decouple from natural hydrologic regimes through managed irrigation inputs [52]. However, double-season crops exhibited nonlinear variations in climatic factor importance, reflecting crop-specific physiological responses. Winter wheat showed temperature sensitivity during dormancy but shifted to water dependence during spring growth, whereas summer maize demonstrated peak soil moisture demand during its summer reproductive phase [57]. This complexity demonstrated that human activities, such as crop rotation and irrigation, can disrupt natural climate control in agroecosystems, causing large and irregular changes in the importance of temperature and moisture.

The variation in importance also differed across vegetation types when temperature and soil moisture served as the independent variable (Figure 8). As temperature increased, its importance generally decreased while that of soil moisture increased, especially in forests, grasslands, and single-crop systems (Figure 8a–d). When soil moisture was the independent variable, no distinct pattern emerged, likely because soil moisture was rarely limited in most areas (Figure 8e–h). These observed differences reflected the distinct optimal growth temperatures and ecological adaptation strategies of each vegetation type [58]. In forests, abundant soil moisture contributed to a balance between temperature and moisture importance near the optimal growth temperature. For grasslands and crops, soil moisture became more influential at higher temperatures due to relatively lower water availability. These patterns underscored how differences in water and temperature sensitivity are closely tied to the adaptive characteristics of each vegetation type [59].

The feature contribution map of IMV-LSTM clarified the role of water–thermal coupling in vegetation growth. However, it did not provide mechanistic insights into vegetation physiological and ecological processes. Moreover, while the reliability and consistency of MODIS LAI products have been extensively validated against ground-based observations across various ecosystems globally, showing good agreement [60,61,62,63], and the reprocessed LAI data utilized in this study has further improved accuracy and reduced inherent uncertainties compared to raw MODIS LAI through advanced algorithms [38], inherent uncertainties within the input data sources, such as known issues with MODIS LAI due to atmospheric interference and potential biases in GLDAS soil moisture over arid/semi-arid regions [64,65,66], further limited the comprehensive understanding. The balance between model complexity and interpretability also limited the choice of model architecture and input variables in this study. For example, other important drivers of vegetation dynamics were omitted to isolate the effects of soil moisture and temperature. Future research could combine process-based models with deep learning frameworks. One possible direction is to embed physical constraints into neural networks or use deep learning to improve results from process-based models. Improved interpretability methods are also necessary. These approaches may help deep learning enhance model accuracy and support more comprehensive simulations of vegetation dynamics under changing environmental conditions.

## 5. Conclusions

This study used three deep learning models (ANN, LSTM, and IMV-LSTM) to simulate LAI in the Loess Plateau, with temperature and soil moisture as the input factors. IMV-LSTM outperformed traditional models, accurately simulating different vegetation types and capturing the bimodal LAI pattern in double-season crops. The attention mechanism showed that the importance of temperature and soil moisture varied across space, season, and vegetation types. Specifically, soil moisture played a dominant role in the arid northwest and during spring and summer growing seasons. In contrast, temperature exerted a stronger influence in the humid southeast and during autumn and winter. Furthermore, distinct response patterns were observed across different vegetation types, highlighting the nuanced interactions. This study demonstrated that the IMV-LSTM model can effectively capture the spatiotemporal dynamics of vegetation–climate interactions and revealed distinct thermal and hydrological regulatory mechanisms across different ecosystems. However, the current model also has limitations. Our model focused mainly on temperature and soil moisture, without comprehensively integrating other environmental variables (e.g., precipitation, solar radiation) and explicit physiological processes. Future research should thus explore incorporating a broader range of variables and integrating process-based models to enhance the mechanistic understanding and predictive accuracy of LAI dynamics.

## Data Availability

Data are available in a publicly accessible repository. The data presented in this study are openly available: The LAI dataset is openly available at http://globalchange.bnu.edu.cn/research/laiv061 (accessed on 24 June 2025). The GLDAS dataset is openly available at https://disc.gsfc.nasa.gov/datasets/GLDAS_NOAH025_3H_2.1/summary (accessed on 24 June 2025). The Landcover dataset is openly available at https://www.earthdata.nasa.gov/data/catalog/lpcloud-mcd12q1-061 (accessed on 24 June 2025).
